# Menopausal wellbeing: navigating quality of life and osteoporosis risk

**DOI:** 10.3389/fpubh.2024.1343160

**Published:** 2024-05-28

**Authors:** Rajeesh R. Nair, Teena Mary Joy, Leyanna Susan George, Aparna Ajay, Minu Maria Mathew, Greeshma C. Raveendran

**Affiliations:** ^1^Department of Community Medicine, Amrita Institute of Medical Sciences, Amrita Vishwa Vidyapeetham, Kochi, India; ^2^Indian Council of Medical Research, New Delhi, India

**Keywords:** postmenopausal women, quality of life, osteoporosis, vasomotor, physical, psychosocial

## Abstract

**Background:**

Multifaceted dimensions influence the quality of life among post-menopausal women. Osteoporosis, a condition characterized by fragile bones, poses a significant risk, potentially leading to fractures and decreased wellbeing. This study aims to assess the quality of life of postmenopausal women, its determinants, and also the risk of osteoporosis among them.

**Methods:**

A cross-sectional study was done among 379 post-menopausal women residing in rural and urban areas of Ernakulam district, Kerala, India. They were selected by probability proportional to size sampling from 10 clusters. Quality of life was measured using MENQOL-I questionnaire and osteoporosis risk assessment was done using OSTA score.

**Results:**

The study participants had a mean age of 60 years, (standard deviation of 6.83 years). On average, menopause occurred at 50.58 years (standard deviation of 4.28 years). The most common symptoms impacting quality of life among postmenopausal women were psychosocial symptoms, followed by physical and vasomotor symptoms. Furthermore, a high proportion (63.6%) of participants were at risk for osteoporosis. History of fracture, concern of falling, marital status and having an insurance, are factors associated with various domains of quality of life.

**Conclusion:**

This study underscores the complex interplay of demographic factors, menopausal experiences, and their impact on the participants' quality of life. The prevalence of psychosocial symptoms and the significant risk of osteoporosis call for tailored healthcare interventions. Postmenopausal women with history of fracture, high concern of fall and single women require special attention. Encouraging women to take up selfcare practices will help during the menopausal transition to have a good quality of life.

## 1 Introduction

Women experience distinct reproductive life stages, including infancy, childhood, adolescence/menarche, adulthood, pre-menopause, menopause, and post-menopause. The World Health Organization (WHO) defines menopause as “a natural phenomenon that is deemed to have occurred after 12 consecutive months without menstruation for which there is no other obvious physiological or pathological cause and in the absence of clinical intervention” (1). During menopause, hormonal changes occur due to decreased ovarian endocrine activity, impacting the production of estrogen and progesterone, potentially affecting quality of life. Menopausal symptoms may encompass vasomotor issues like hot flashes and night sweats, psycho-social challenges including increased anxiety and reduced sleep, physical discomfort like joint and knee pain, backache, and vaginal dryness, as well as reduced interest in sexual activity. These and the related changes occurring in cardiovascular risk profile and osteoporosis are well-elucidated in the SWAN study (2). Numerous global studies have explored the quality of life in menopausal women, employing various assessment tools, with the Menopause-Specific Quality of Life Questionnaire-Intervention (MENQOL-I) (3) being a commonly used instrument. Studies have shown that women at early peri, late peri or post menopause exhibited significant reduction in functioning than premenopausal women (4). National level studies have shown prevalence of post-menopausal women experiencing menopausal symptoms and poor quality of life to range between 70.2 and 73.8% (5, 6).

The concept of QoL encompasses various dimensions, including physical, psychological, and social wellbeing, as well as functional status. Osteoporosis can profoundly impact each of these dimensions, compromising an individual's ability to engage in daily activities, maintain independence, and enjoy a fulfilling life. Postmenopausal osteoporosis (PMO) is a generalized skeletal disorder in which decreased bone density contributes to increased risk of fracture (7). A hospital-based study from North India, estimated the prevalence of osteoporosis to be 37.5% among postmenopausal women (8).

Standard equipment used to measure osteoporosis is the use of Dual Energy Xray Absorptiometry (DEXA) scan which is expensive, inaccessible and makes use of high energy X-Ray radiation. One low cost, easy, no radiation tool for screening osteoporosis in older people is Osteoporosis Self-Assessment Tool for Asians (OSTA) (9).

One of the causes of fracture is minor trauma that occurs following falls. Older people with past history of falls have been found to have increased risk of falls. A tool that is used to assess the fear of fall in older people is the Fall Efficacy Scale International (FES-I) questionnaire FES-I.^1^

This study aims to assess the quality of life and its determinants of post-menopausal women in Ernakulam district using the menopause specific Quality of Life Intervention (MENQOL-I) scale. This study also tries to look at risk of osteoporosis among menopausal women, and fear of fall as they are factors that contribute to fragility fractures and increased morbidity among older people. These measures are now in focus since Kerala is experiencing demographic transition in which there is an increase in the number of people in older age groups, especially women.

## 2 Methods

### 2.1 Participants

A community-based cross-sectional study was conducted in Ernakulam district, Kerala state, situated in the central region with a population of 3.2 million. The district comprises of seven talukas, including one corporation, 11 municipalities, and 14 block panchayaths. The basic unit was the ward in rural areas, with a population of 1,000–1,500 per ward and division in urban areas, each division with 7,500–10,000 people. These basic units were considered as clusters. Ten clusters were selected using probability proportional to size sampling from these administrative divisions. The chosen clusters were Parakkadavu, Rayamangalam, Choornikkara, Thuravoor, Eloor, Mulanthuruthy, Ramamangalam, Vytilla, Karuvelippady, and Palarivattom, covering both rural and urban areas.

The study focused on women who had completed 12 months without menstruation, excluding those over 70 years old because as age increases the physiological changes due to aging process will also affect the quality of life and those who reached menopause through surgical interventions like hysterectomy or bilateral oophorectomy. The sample size was determined based on a study by Pathak and Shivaswamy, with a prevalence of 63.9% for psychosocial symptoms and a relative precision of 10%, resulting in a sample size of 374 after applying a design effect of 1.5 (10).

The number of households to be visited in each cluster (ward) was calculated, and it was determined to be 38 post-menopausal women per cluster (Sample size/number of clusters). In cases where residents were not available during the initial visit, households were revisited at least twice, and data were collected.

For participant selection, starting from the first house to the right side of the first by-lane, adjacent houses were visited until 38 eligible females who had not experienced menstruation in the last 12 months and provided informed consent, were enrolled. This process was repeated for all 10 clusters.

### 2.2 Ethical considerations

The protocol designed for the present study was submitted to the Amrita School of Medicine Ethical Committee from where ethical clearance certificate (ECASM-AIMS-2021-008) was issued before the start of the study. The consent was printed in Malayalam, the local language and details of the study were explained to the participants. Written informed consent was obtained prior to data collection.

### 2.3 Variables and measurement

#### 2.3.1 Study variables

Using a standard questionnaire and equipment following demographic and anthropometric measures were taken: age (years), age of attaining menopause (years), height (m), weight (Kg), Body Mass Index (BMI) (Kg/m^2^), education, occupation, religion, area of residence, ownership of house, marital status, type of family, health care expense, socioeconomic status based on color of ration (public distribution system) card, history of fracture bone, calcium supplementation, calcium rich food intake.

#### 2.3.2 Study tools

##### 2.3.2.1 Menopause specific quality of life-intervention (MENQOL-I) questionnaire

Permission to use MENQOL-I questionnaire was obtained online from the e-Provide-Mapi Trust organization (11). The instrument assesses quality of life across four domains: Vasomotor, Psychosocial, Physical, and Sexual domains. The questionnaire was administered through interviews. Participants answered “No” if no symptoms were present. If they responded affirmatively, they were asked to assign scores from 0 to 6, indicating the degree of botheration caused by the symptom (0 = not at all bothered to 6 = extremely bothered. The scores were then converted to conversion scores as follows: “No” is taken as 1, “Yes”: 0 = 2, 1 = 3, 2 = 4, 3 = 5, 4 = 6, 5 = 7, and 6 = 8, for purpose of analysis.

Mean scores for each domain were calculated as the sum of scores in each domain divided by the number of symptoms in that domain. The Likert responses were categorized based on mean scores as either Low symptom score or High symptom score, following the methodology outlined by Bhandari (12). After determining the mean score in each domain, those above the mean score were classified as having a high symptom score, while those below the mean were designated as having a low symptom score.

Since some of the participants were from rural areas and had difficulty in reading the MENQOL-I questionnaire, the same was administered by interview method. In rural areas and houses with large families, the administration of the questionnaire was done in the presence of other family members. So, it was difficult for the data collector to obtain information about responses in the sexual domain.

##### 2.3.2.2 Osteoporosis self-assessment tool for Asians

This tool can be used for calculation of risk of osteoporosis based on formula: [weight (Kg)-age (years)] × 0.2 (9). Based on the score, risk of osteoporosis is calculated as Mild (>-1), Intermediate (score −1 to −4) and High risk (score < −4) of osteoporosis.

##### 2.3.2.3 Fall Efficacy Scale International questionnaire (FES-I)

This questionnaire was used for assessing the fear of fall among older adult postmenopausal women. It has 16 questions answered with scores 1–4 by the woman. Based on the results, fear of fall is assessed as mild concern (16–19), moderate concern (20–27) and high concern (28–64) of fall.

### 2.4 Statistics

The data were tabulated on MS Excel sheet. Statistical Analysis was done with IBM SPSS Statistics for Windows, Version 21.0. Armonk, NY: IBM Corp. Quantitative variables with normal distribution were expressed as mean and standard deviations. Quantitative variables which were not normally distributed were expressed as median and interquartile range. Categorical variables were expressed as frequency and percentages.

Chi-square test was used to test association of various factors with designated high symptom score and low symptom score of quality of life. After logistic regression, all determinants with a *p*-value < 0.05 was assumed to be independent predictors of quality of life in its various domains (**Table 6**).

## 3 Results

The average age of the 379 post-menopausal women in this study was 60 years, with a standard deviation of 6.83 years. The mean age at menopause was determined to be 50.58 years, with a standard deviation of 4.28 years. Post-menopausal women in the study had an average BMI of 24.56 kg/m^2^.

The majority of study participants identified with the Hindu religion (59.1%), while 34% had attained a high school level of education. Homemakers constituted 89.2% of the participants, and 55.4% resided in nuclear families. Majority, 89.4%, were married. Urban residents accounted for 50.4% of the participants, and 83.6% lived in self-owned houses. Notably, 95% reported Out-Of-Pocket expenses in healthcare, and 63.8% belonged to Above Poverty Line (APL) families based on their ration/public distribution system cards.

Among the post-menopausal women, 55.4% were classified as overweight, and 15.3% were categorized as obese. Additionally, 12.9% had a history of bone fractures, 22.4% were on calcium supplementation, and 35.4% reported a history of consuming calcium-rich foods such as milk and green leafy vegetables (Table 1).

**Table 1:** Distribution of sociodemographic and other determinants among post-menopausal women in Ernakulam district.

**Characteristics**	**Category**	**Frequency (*n* = 379)**	**%**
**Sociodemographic determinants**			
Age	≤ 60 years	202	53.3
	>60 years	177	46.7
Age of attaining menopause	≤ 50 years	214	56.5
	>50 years	165	43.5
Religion	Hindu	224	59.1
	Christian	99	26.1
	Muslim	56	14.8
Education	Primary	64	16.9
	Middle	124	32.7
	High school	129	34.0
	Higher secondary	45	11.9
	Graduation and above	17	4.5
Occupation	Unskilled	20	5.3
	Skilled	9	2.4
	Professional	2	0.5
	Home maker	338	89.2
	Retired	10	2.6
Type of family	Nuclear family	210	55.4
	Joint Family	12	3.2
	3 generation family	157	41.4
Marital status	Married	339	89.4
	Unmarried	3	0.8
	Widow	37	9.8
Area of residence	Rural	188	49.6
	Urban	191	50.4
Type of house	Owned	317	83.6
	Rent	62	16.4
Health care expenses	Out-of-pocket (OOP) expenses	360	95
	Insurance	19	5
Socio-economic status based on Ration card	BPL	137	36.2
	APL	242	63.8
BMI (South-east Asian)	Underweight	15	4.0
	Normal weight	96	25.3
	Overweight	210	55.4
	Obese	58	15.3
**Other determinants**			
History of fracture bone	Yes	49	12.9
	No	330	87.1
Calcium supplementation	Yes	85	22.4
	No	294	77.6
Calcium rich food	Yes	134	35.4
	No	245	64.6
Risk of osteoporosis based on OSTA score	Mild risk of osteoporosis	138	36.4
	Moderate risk of osteoporosis	201	53.0
	High risk osteoporosis	40	10.6
Fear of fall (based on fall efficacy Scale International)	Low concern about fear of fall	225	59.4
	Moderate concern about fear of fall	109	28.8
	High concern about fear of fall	45	11.9

BPL, below poverty line; APL, above poverty line; BMI, body mass index; OSTA, Osteoporosis Self-assessment Tool for Asians.

According to responses from the MENQOL-I questionnaire, 27% of women (103) reported experiencing hot flashes in the vasomotor domain. In the psychosocial domain, 26.6% (101) mentioned accomplishing less than they used to. For the physical domain, 64.6% (245) of post-menopausal women reported aching muscles and joints (Table 2).

**Table 2:** Distribution of post-menopausal women based on response to MENQOL-I questionnaire.

**S.no**	**MENQOL-I**	**Answered yes Frequency (*n* = 379)**	**Percentage**
**I**.	Vasomotor domain		
1	Hot flushes or flashes	103	27.2
2	Night sweats	62	16.4
3	Sweating	99	26.1
**II**.	Psycho-social domain		
4	Dissatisfaction with my personal life	54	14.2
5	Feeling anxious or nervous	138	36.4
6	Poor memory	33	8.7
7	Accomplishing less than I used to	101	26.6
8	Feeling depressed, down or blue	24	6.3
9	Being impatient with other people	86	22.7
10	Feeling of wanting to be alone	6	1.6
**III**.	Physical domain		
11	Flatulence (wind) or gas pains	44	11.6
12	Aching in muscles and joints	245	64.6
13	Feeling tired and worn out	139	36.7
14	Difficulty sleeping	84	22.2
15	Aches in back of neck or head	142	37.5
16	Decrease in physical strength	79	20.8
17	Decrease in stamina	51	13.5
18	Lack of energy	92	24.3
19	Dry skin	75	19.8
20	Weight gain	92	24.3
21	Increased facial hair	41	10.8
22	Changes in appearance, texture or tone of your skin	27	7.1
23	Feeling bloated	40	10.6
24	Low backache	103	27.2
25	Frequent urination	38	10
26	Involuntary urination while laughing or coughing	14	3.7
30	Breast pain or tenderness	23	6.1
31	Vaginal bleeding or spotting	36	9.5
32	Leg pains or cramps	191	50.4

**Figure 1:**
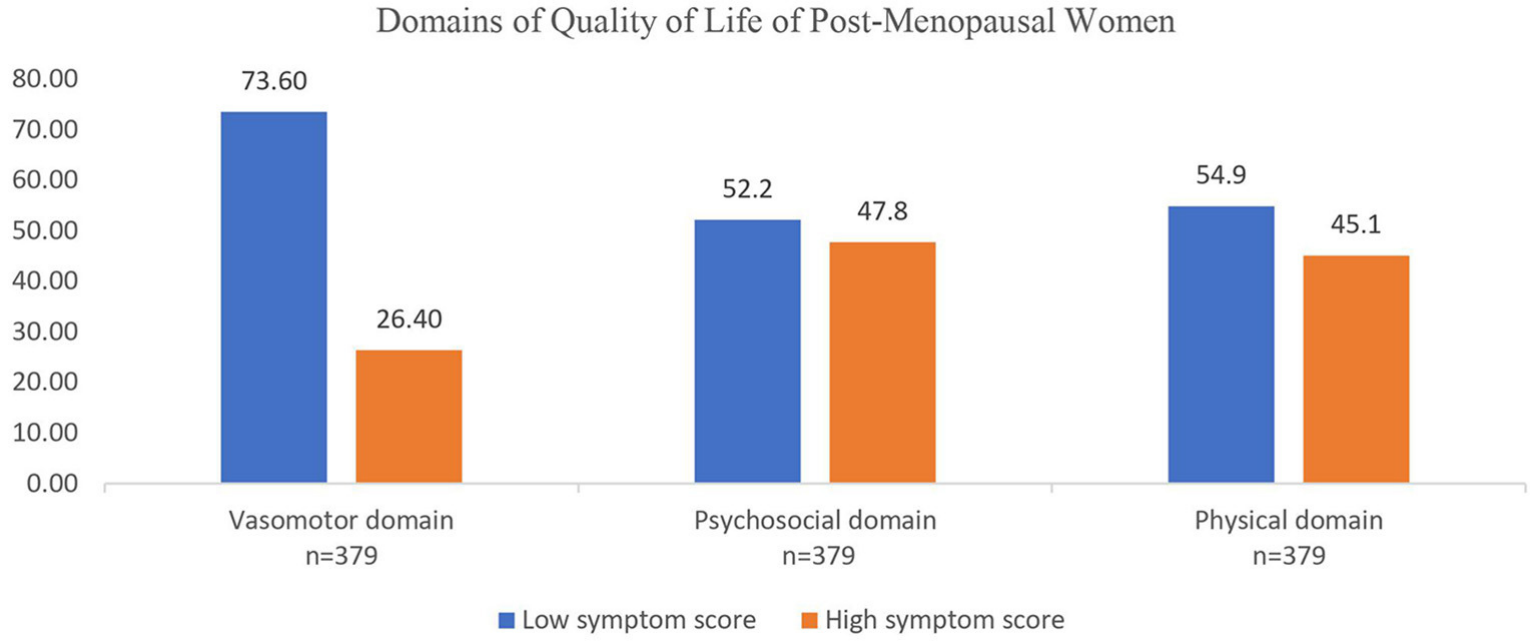
Domains of quality of life of post-menopausal women based in symptom score.

The psychosocial domain exhibited the highest impact on postmenopausal women, affecting 47.8% (181) of them, suggesting that this domain had the most significant influence. In close succession, the physical domain impacted 45.1% (171) of postmenopausal women, whereas the vasomotor domain had the lowest proportion, with 26.4% (100) of postmenopausal women affected (Figure 1).

Participants in the study perceived psychosocial symptoms such as anxiety or nervousness (36.4%) and impatience with others (22.7%) as highly impactful on their quality of life. Additionally, they identified physical symptoms like muscle and joint aches (64.6%), discomfort in the back of the neck and head (37.5%), and feelings of tiredness and fatigue (36.7%) as affecting their quality of life. Notably, a smaller proportion of women perceived vasomotor symptoms such as hot flashes (27.2%), sweating (26.1%), and night sweats (16.4%) as impacting their quality of life.

The various socio-demographic factors, risk of osteoporosis based on OSTA score, fear of fall based on Fall Efficacy Scale International questionnaire were checked for association with impact score in quality of life (high and low) in the vasomotor, physical and psychosocial domains. This analysis is represented in the Tables 3–5. All factors with a *p*-value in chi-square test < 0.2 were further used in the logistic regression modeling, backward conditional model was used.

**Table 3:** Results of univariate analysis to test association of determinant factors on Vasomotor domain of MENQOL-I questionnaire.

**Variables**	**Categories**	**Symptoms of postmenopausal women in vasomotor domain**		**Pearsons Chi-square test**	***p*-value**
		**Less impact**	**High impact**		
Age	≤ 60 years *n* = 202	141 (69.8)	61 (30.2)	3.24	**0.072**
	>60 years *n* = 177	138 (78.0)	39 (22.0)		
Age of menopause	≤ 50 years *n* = 214	156 (72.9)	58 (27.1)	0.13	0.718
	>50 years *n* = 165	123 (74.5)	42 (25.5)		
Area of residence	Rural *n* = 188	140 (74.5)	48 (25.5)	0.14	0.708
	Urban *n* = 191	139 (72.8)	52 (27.2)		
House of residence	Owned *n* = 317	232 (73.2)	85 (26.8)	0.67	0.754
	Rent *n* = 62	47 (75.8)	15 (24.2)		
Religion	Hindu *n* = 224	172 (76.8)	52 (23.2)	4.76	0.092
	Christian *n* = 99	72 (72.7)	27 (27.3)		
Muslim *n* = 56	35 (62.5)	21 (37.5)		
Education^*^	Pre-high school *n* = 188	136 (72.3)	52 (27.7)	1.75	0.417
	High school *n* = 129	100 (77.5)	29 (22.5)		
After high school *n* = 62	43 (69.4)	19 (30.6)		
Occupation^**^	Employed *n* = 41	24 (58.5)	17 (41.5)	5.38	**0.020**
	Home maker *n* = 338	255 (75.4)	83 (24.6)		
Socioeconomic status^***^	BPL *n* = 137	99 (72.3)	38 (27.7)	0.20	0.653
	APL *n* = 177	180 (74.4)	62 (25.6)		
Marital status^#^	Married n = 339	251 (74.0)	88 (26.0)	0.30	0.583
	Single *n* = 40	28 (70.0)	12 (30.0)		
Type of family^##^	Nuclear *n* = 210	150 (71.4)	60 (28.6)	1.16	0.282
	Extended *n* = 169	129 (76.3)	40 (23.7)		
Payment of health care expenses	Out of pocket expenses *n* = 360	269 (74.7)	91 (25.3)	4.53	**0.057**
	Insurance or other support *n* = 19	10 (52.6)	9 (47.4)		
BMI (South east Asian)^###^	Low BMI *n* = 111	82 (73.9)	29 (26.1)	0.01	1.000
	High BMI *n* = 268	197 (71)	71 (26.5)		
Risk of osteoporosis based^@^	Low risk of osteoporosis *n* = 138	95 (68.8)	43 (31.2)	2.457	**0.12**
	High risk of osteoporosis *n* = 241	184 (76.3)	57 (23.7)		
Fear of fall^@@^	Low concern of falls *n* = 225	169 (75.1)	56 (24.9)	0.638	0.48
	High concern of falls *n* = 154	110 (71.4)	44 (28.6)		

The bold values are the factors which have been considered for logistic regression analysis. ^*^Education: Pre high school (Primary, Middle school), High School, Post high school (Secondary, Higher secondary, Graduation). ^**^Occupation: Employed (Unskilled, skilled, professional, retired); Homemaker. ^***^Socioeconomic: BPL (Yellow, pink); APL (Blue, white). ^#^Marital status: Married, Single (Unmarried, widow). ^##^Type of family: Nuclear, Extended (Joint family, three generation family). ^###^BMI South-east Asian: Low BMI (Underweight, normal weight); high BMI (Overweight, obese). ^@^Risk of osteoporosis: Low risk, High risk (Moderate risk, High risk). ^@@^Concern about falling: Low concern, High concern (moderate concern, high concern).

**Table 4:** Results of univariate analysis to test association of determinant factors on psychosocial domain of MENQOL-I questionnaire.

**Variables**	**Categories**	**Symptoms of postmenopausal women in psychosocial domain**		**Pearsons Chi-square test**	***p*-value**
		**Low impact**	**High impact**		
Age	≤ 60 years *n* = 202	117 (57.9)	85 (42.1)	5.59	**0.023**
	>60 years *n* = 177	81 (45.8)	96 (54.2)		
Age of menopause	≤ 50 years *n* = 214	120 (56.1)	94 (43.9)	2.89	**0.098**
	>50 years *n* = 165	78 (47.3)	87 (52.7)		
Area of residence	Rural *n* = 188	93 (49.5)	95 (50.5)	1.15	0.305
	Urban = 191	105 (55.0)	86 (45.0)		
House of residence	Owned *n* = 317	164 (51.7)	153 (48.3)	0.20	0.679
	Rent *n* = 62	34 (54.8)	28 (45.2)		
Religion	Hindu *n* = 222	123 (54.9)	101 (45.1)	1.65	0.437
	Christian *n* = 99	47 (47.5)	52 (52.5)		
Muslim *n* = 56	28 (50.0)	28 (50.0)		
Education^*^	Pre-high school *n* = 188	95 (50.5)	93 (49.5)	4.56	**0.102**
	High school *n* = 129	63 (48.8)	66 (51.2)		
After high school *n* = 62	40 (64.5)	22 (35.5)		
Occupation^**^	Employed *n* = 41	24 (58.5)	17 (41.5)	0.73	0.413
	Homemaker n = 228	174 (51.5)	164 (48.5)		
Socioeconomic status^***^	BPL *n* = 137	66 (48.2)	71 (51.8)	1.42	0.241
	APL *n* = 142	132 (54.5)	110 (45.5)		
Marital status^#^	Married *n* = 339	179 (52.8)	160 (47.2)	0.40	0.616
	Single *n* = 40	19 (47.5)	21 (52.5)		
Type of family^##^	Nuclear *n* = 210	116 (55.2)	94 (44.8)	1.69	0.215
	Extended *n* = 169	82 (48.5)	87 (51.5)		
Payment of health care expenses	Out of Pocket *n* = 360	192 (53.3)	168 (46.7)	3.42	**0.097**
	Insurance or other support *n* = 19	6 (31.6)	13 (68.4)		
BMI (Southeast Asian)^###^	Low BMI *n* = 111	55 (49.5)	56 (50.5)	0.46	0.572
	High BMI *n* = 268	143 (53.4)	125 (46.6)		
History of fracture bone	Yes *n* = 49	16 (32.7)	33 (67.3)	8.66	**0.004**
	No *n* = 330	182 (55.2)	148 (44.8)		
Calcium supplementation	Yes *n* = 85	45 (52.9)	40 (47.1)	0.02	0.902
	No *n* = 294	153 (52)	141 (48.0)		
Calcium rich food	Yes *n* = 134	68 (50.7)	66 (49.3)	0.19	0.669
	No *n* = 245	130 (53.1)	115 (46.9)		
Risk of osteoporosis based^@^	Low risk of osteoporosis *n* = 138	72 (52.2)	6 (47.8)	< 0.001	1.000
	High risk of osteoporosis *n* = 241	126 (52.3)	115 (47.7)		
Concern of fall^@@^	Low concern of falls *n* = 225	140 (62.2)	85 (37.8)	22.103	**< 0.001**
	High concern of falls *n* = 154	58 (37.7)	96 (62.3)		

The bold values are the factors which have been considered for logistic regression analysis. ^*^Education: Pre high school (Primary, Middle school), High School, Post high school (Secondary, Higher secondary, Graduation). ^**^Occupation: Employed (Unskilled, skilled, professional, retired); Homemaker. ^***^Socioeconomic: BPL (Yellow, pink); APL (Blue, white). ^#^Marital status: Married, Single (Unmarried, widow). ^##^Type of family: Nuclear, Extended (Joint family, three generation family). ^###^BMI South-east Asian: Low BMI (Underweight, normal weight); high BMI (Overweight, obese). ^@^Risk of osteoporosis: Low risk, High risk (Moderate risk, High risk). ^@@^Concern about falling: Low concern, High concern (moderate concern, high concern).

**Table 5:** Results of univariate analysis to test association of determinant factors on physical domain of MENQOL-I questionnaire.

**Variables**	**Categories**	**Symptoms of postmenopausal women in physical domain**		**Pearsons Chi-square test**	***p*-value**
		**High impact**	**Low impact**		
Age	≤ 60 years *n* = 202	123 (60.9)	79 (39.1)	6.31	**0.13**
	>60 years *n* = 177	85 (48)	92 (52.0)		
Age of menopause	≤ 50 years *n* = 214	121 (56.5)	93 (43.5)	0.55	0.468
	>50 years *n* = 165	87 (52.7)	78 (47.3)		
Area of residence	Rural *n* = 188	97 (51.6)	91 (48.4)	1.63	0.216
	Urban *n* = 191	111 (58.1)	80 (41.9)		
House of residence	Owned *n* = 317	181 (57.1)	136 (42.9)	3.85	**0.052**
	Rent *n* = 62	27 (43.5)	35 (56.5)		
Religion	Hindu *n* = 224	119 (53.1)	105 (46.9)	3.68	0.159
	Christian *n* = 99	62 (62.6)	37 (37.4)		
Muslim *n* = 56	27 (48.2)	29 (51.8)		
Education^*^	Pre-high school *n* = 188	99 (52.7)	89 (47.3)	2.01	0.367
	High school *n* = 129	70 (54.3)	59 (45.7)		
After high school *n* = 62	39 (62.9)	23 (37.1)		
Occupation^**^	Employed *n* = 41	21 (51.2)	20 (48.8)	0.25	0.623
	Homemaker *n* = 338	187 (55.3)	151 (44.7)		
Socioeconomic status^***^	BPL *n* = 111	54 (48.6)	57 (51.4)	2.46	**0.140**
	APL *n* = 268	154 (57.5)	114 (42.5)		
Marital status^#^	Married *n* = 339	196 (57.8)	143 (42.2)	11.18	**0.001**
	Single *n* = 40	12 (30.0)	28 (70.0)		
Type of family^##^	Nuclear *n* = 210	124 (59.0)	86 (4.0)	3.30	**0.078**
	Extended *n* = 169	84 (49.7)	85 (50.3)		
Payment of health care expenses	Out of Pocket *n* = 360	197 (54.7)	163 (45.3)	0.07	0.818
	Insurance or other support n = 19	11 (57.9)	8 (42.1)		
BMI (Southeast Asian)^###^	Low bmi *n* = 111	54 (48.6)	57 (51.4)	2.46	**0.140**
	High bmi *n* = 268	154 (57.5)	114 (42.5)		
History of fracture bone	Yes *n* = 49	21 (42.9)	28 (57.1)	3.28	**0.090**
	No *n* = 330	187 (56.7)	143 (43.3)		
Calcium supplementation	Yes *n* = 85	41 (48.2)	44 (51.8)	1.96	**0.175**
	No *n* = 294	167 (56.8)	127 (43.2)		
Calcium rich food	Yes *n* = 134	70 (52.2)	64 (47.8)	0.59	0.452
	No *n* = 245	138 (56.3)	107 (43.7)		
Risk of osteoporosis based^@^	Low risk of osteoporosis *n* = 138	75 (54.3)	63 (45.7)	0.025	0.915
	High risk of osteoporosis *n* = 241	133 (55.2)	108 (44.8)		
Concern of fall^@@^	Low concern of falls *n* = 225	161 (71.6)	64 (28.4)	62.175	**< 0.001**
	High concern of falls *n* = 154	47 (30.5)	107 (69.5)		

The bold values are the factors which have been considered for logistic regression analysis. ^*^Education: Pre high school (Primary, Middle school), High School, Post high school (Secondary, Higher secondary, Graduation). ^**^Occupation: Employed (Unskilled, skilled, professional, retired); Homemaker. ^***^Socioeconomic: BPL (Yellow, pink); APL (Blue, white). ^#^Marital status: Married, Single (Unmarried, widow). ^##^Type of family: Nuclear, Extended (Joint family, three generation family). ^###^BMI South-east Asian: Low BMI (Underweight, normal weight); high BMI (Overweight, obese). ^@^Risk of osteoporosis: Low risk, High risk (Moderate risk, High risk). ^@@^Concern about falling: Low concern, High concern (moderate concern, high concern).

Logistic regression analysis was conducted to identify determinants affecting quality of life domains. In the vasomotor domain, occupation (employed) showed a significant association [adjusted odds ratio (aOR): 2.07, 95% confidence interval (C.I.): 1.05, 4.09]. In the psychosocial domain, factors such as age of attaining menopause (>50 years, aOR: 1.54, 95% C.I.: 1.00, 2.37), payment of health expenses (insurance, aOR: 3.24, 95% C.I.: 1.16, 9.00), history of bone fracture (yes, aOR: 2.34, 95% C.I.: 1.21, 4.52), and concern of falling (high concern, aOR: 2.74, 95% C.I.: 1.78, 4.23) were statistically significant. In the physical domain, factors such as house ownership (rent, aOR: 1.84, 95% C.I.: 1.42, 6.69), marital status (single, aOR: 3.09, 95% C.I: 1.42, 6.69), and concern of falling (high concern, aOR: 5.49, 95% C.I.: 3.48, 8.66) were found to be statistically significant (Table 6).

**Table 6:** Logistic regression analysis for identifying the determinants of post-menopausal symptoms under various domains of MENQOL-I questionnaire.

**Variable**	**Categories**	**OR (95% C.I)**	***p*-value**
**Vasomotor domain**			
Occupation	Homemaker (ref)	1	0.040
	Employed	2.07 (1.05, 4.09)	
**Psychosocial domain**			
Age of menopause	≤ 50 years (ref)	1	
	>50 years	1.54 (1.00, 2.37)	0.048
Payment of health care expenses	OOPE (ref)	1	
	Insurance	3.24 (1.164, 9.00)	0.024
History of fracture bone	No (ref)	1	
	Yes	2.34 (1.21, 4.52)	0.011
Concern of fall	Low concern (ref)	1	
	High Concern	2.74 (1.78, 4.23)	< 0.001
**Physical domain**			
Type of house	Owned (ref)	1	
	Rent	1.84 (1.06, 3.21)	0.03
Marital status	Married (ref)	1	
	Single	3.09 (1.42, 6.69)	0.004
Concern of fall	Low concern (ref)	1	
	High concern	5.49 (3.48, 8.66)	< 0.001

OR, odds ratio; 95% CI, 95 percent confidence interval; OOPE, out of pocket expenses.

The evaluation of osteoporosis risk among post-menopausal women revealed that more than half (53%) of the post-menopausal women had moderate risk while 10.6% (40) were at high risk and 36.4% (138) at mild risk, respectively.

## 4 Discussion

In this cross-sectional community-based study involving 379 post-menopausal women from Ernakulam district, Kerala, the quality of life was assessed using the MENQOL-I questionnaire in the Vasomotor, Psychosocial, and Physical domains.

Studies from other parts of the country showed that the symptoms based on domains in MENQOL that affected the quality of life were different from the current study. However, pattern that was prominent were the symptoms in physical domain which affects the quality of life in post-menopausal women. Gaikwad et al. in a study looking at menopausal symptoms among teachers in Raipur, Chhattisgarh, found that 87.5% of the study participants had symptoms in the psychosocial domain, followed by 68.05% participants having symptoms in the physical domain and 51.85% suggesting vasomotor symptoms as affecting the quality of life (13).

Logistic regression analysis indicated that the determinant associated with symptoms in the vasomotor domain was occupation (employed participants). Women with high symptom scores in the psychosocial domain, were those who attained menopause above 50 years, participants with insurance, having a history of fracture and a high concern of falling. In the physical domain, high symptom scores were associated with women living in rented house, single women and those with high concern of falling.

In a study by Karmakar et al. on the quality of life of post-menopausal women in West Bengal, it was found that vasomotor symptoms were associated with type of family; psychological symptoms were associated with age; physical symptoms with caste, education and marital status (14). In a study from Nepal, significant factors that were associated with quality of life were marital status, number of children, education, occupation and health seeking behavior (15). Studies have found women with occupation and higher educational status to have lower scores (16, 17).

In the current study, participants who were employed had higher odds of suffering from vasomotor symptoms. The stress of managing household work and occupation simultaneously may have a bearing on the condition. Many employed women pass through midlife crisis typically at this age. Having a supportive environment both at home and workplace can ease the stress.

Insured participants in this study had higher score in the psychosocial domain, reflecting the fact that better economic circumstances may influence health seeking behavior. Women with history of fracture and concerned about falling also had higher psychosocial domain scores. In another study conducted among menopausal women in urban part of Hyderabad, India characteristics like education and socioeconomic status were significant determinants of quality of life (6). In this study, among postmenopausal women, risk of osteoporosis was high in 40 (10.6%), moderate in 201 (53%) and mild risk seen in 138 (36.4%) of post-menopausal women.

In a study assessing the OSTA tool's efficacy in evaluating the risk of non-vertebral fractures in post-menopausal Chinese women, findings revealed that 46.1% exhibited high-risk osteoporosis, 34.3% showed moderate risk, and 19.6% had low risk (18). Another study by Ha and Baek found an association between BMI and lower bone mineral density, indicating a potential link with osteoporosis (19). Moreover, research by Kung and Huang emphasized genetic and environmental factors as contributors to osteoporosis, with environmental determinants including smoking, alcohol consumption, low calcium intake, low body weight, and physical inactivity (20).

In this community-based study of postmenopausal quality of life among women, it was found that quality of life was affected by multiple determinants. In depth analysis of the real scenario regarding these determinants calls for a mixed methodology approach. Only three domains namely, vasomotor, psychosocial and physical were addressed. The cultural scenario prevalent in the study area made it hard to explore the quality of life in sexual domain. Conversations about menopause are frequently avoided in families, communities, and workplaces. Providing a supportive environment at home and workplace and having a fall safe environment can improve the quality of life of postmenopausal women. Prevention of fractures should also be given importance, since women with a history of fracture had lesser quality of life. In view of the fact that being single aggravates the physical symptoms of menopause; having good friends and family support may contribute to improved quality of life during menopausal transition.

## Data availability statement

The raw data supporting the conclusions of this article will be made available by the authors, without undue reservation.

## Ethics statement

The studies involving humans were approved by Amrita School of Medicine Ethical Committee. The studies were conducted in accordance with the local legislation and institutional requirements. The participants provided their written informed consent to participate in this study.

## Author contributions

RN: Methodology, Software, Conceptualization, Data curation, Formal analysis, Investigation, Project administration, Resources, Writing – original draft. TJ: Methodology, Software, Supervision, Validation, Visualization, Writing – review & editing. LG: Conceptualization, Methodology, Writing – review & editing. AA: Writing – review & editing, Project administration. MM: Data curation, Formal analysis, Writing – review & editing, Software. GR: Data curation, Formal analysis, Writing – review & editing.

## Footnotes

^1^https://sites.manchester.ac.uk/fes-i/ (accessed September 12, 2022).
